# Supplementary material for: Machine learning for predicting antimicrobial resistance in critical and high-priority pathogens: A systematic review considering antimicrobial susceptibility tests in real-world healthcare settings
Source: PLoS One. 2025 Feb 25;20(2):e0319460. doi: 10.1371/journal.pone.0319460 (PMC11856330; doi:10.1371/journal.pone.0319460)
Supplement: S1 Table — (DOCX) [file pone.0319460.s002.docx]

**Supplementary 2. Table**. List of excluded studies with reasons

|  | Articles Information | Exclusion reason | Data  extractor | Date of  extraction |
| --- | --- | --- | --- | --- |
| 1 | Strich JR, et al. Assessing Clinician Utilization of Next-Generation Antibiotics Against Resistant Gram-NCMAative Infections in U.S. Hospitals: A Retrospective Cohort Study. Ann Intern Med. 2024;177(5):559-572. doi: 10.7326/M23-2309. | No antimicrobial susceptibility tests | CMA and DGA | November 2024 |
| 2 | Hur R, Golik S, She Y. Leveraging Large Data, Statistics, and Machine Learning to Predict the Emergence of Resistant E. coli Infections. Pharmacy (Basel). 2024;12(2):53. doi: 10.3390/pharmacy12020053. | No patient data from hospital information systems | CMA and DGA | November 2024 |
| 3 | Choi MH, Kim D, Park Y, Jeong SH. Development and validation of artificial intelligence models to predict urinary tract infections and secondary bloodstream infections in adult patients. J Infect Public Health. 2024;17(1):10-17. doi: 10.1016/j.jiph.2023.10.021. | No antimicrobial susceptibility tests | CMA and DGA | November 2024 |
| 4 | Zhang YM, Tsao MF, Chang CY, Lin KT, Keller JJ, Lin HC. Rapid identification of carbapenem-resistant Klebsiella pneumoniae based on matrix-assisted laser desorption ionization time-of-flight mass spectrometry and an artificial neural network model. J Biomed Sci. 2023;30(1):25. doi: 10.1186/s12929-023-00918-2. | No patient data from hospital information systems | CMA and DGA | November 2024 |
| 5 | Yu J, Lin YT, Chen WC, Tseng KH, Lin HH, Tien N, Cho CF, Huang JY, Liang SJ, Ho LC, Hsieh YW, Hsu KC, Ho MW, Hsueh PR, Cho DY. Direct prediction of carbapenem-resistant, carbapenemase-producing, and colistin-resistant Klebsiella pneumoniae isolates from routine MALDI-TOF mass spectra using machine learning and outcome evaluation. Int J Antimicrob Agents. 2023;61(6):106799. doi: 10.1016/j.ijantimicag.2023.106799. | No patient data from hospital information systems | CMA and DGA | November 2024 |
| 6 | Chung CR, Wang HY, Yao CH, Wu LC, Lu JJ, Horng JT, Lee TY. Data-Driven Two-Stage Framework for Identification and Characterization of Different Antibiotic-Resistant Escherichia coli Isolates Based on Mass Spectrometry Data. Microbiol Spectr. 2023;11(3):e0347922. doi: 10.1128/spectrum.03479-22. | No patient data from hospital information systems | CMA and DGA | November 2024 |
| 7 | Abu-Aqil G, Suleiman M, Sharaha U, Riesenberg K, Lapidot I, Huleihel M, Salman A. Fast identification and susceptibility determination of E. coli isolated directly from patients' urine using infrared-spectroscopy and machine learning. Spectrochim Acta A Mol Biomol Spectrosc. 2023;285:121909. doi: 10.1016/j.saa.2022.121909. | No patient data from hospital information systems | CMA and DGA | November 2024 |
| 8 | Suleiman M, Abu-Aqil G, Sharaha U, Riesenberg K, Lapidot I, Salman A, Huleihel M. Infra-red spectroscopy combined with machine learning algorithms enables early determination of Pseudomonas aeruginosa's susceptibility to antibiotics. Spectrochim Acta A Mol Biomol Spectrosc. 2022;274:121080. doi: 10.1016/j.saa.2022.121080. | No patient data from hospital information systems | CMA and DGA | November 2024 |
| 9 | Jeon K, Kim JM, Rho K, Jung SH, Park HS, Kim JS. Performance of a Machine Learning-Based Methicillin Resistance of Staphylococcus aureus Identification System Using MALDI-TOF MS and Comparison of the Accuracy according to SCCmec Types. Microorganisms. 2022;10(10):1903. doi: 10.3390/microorganisms10101903. | No patient data from hospital information systems | CMA and DGA | November 2024 |
| 10 | B. Cánovas-Segura et al., Improving Interpretable Prediction Models for Antimicrobial Resistance, 2019 IEEE 32nd International Symposium on Computer-Based Medical Systems (CBMS), Cordoba, Spain, 2019, pp. 543-546, doi: 10.1109/CBMS.2019.00111. | No patient data from hospital information systems | CMA and DGA | November 2024 |
| 11 | Alawieh A, Sabra Z, Bizri AR, Davies C, White R, Zaraket FA. A computational model to monitor and predict trends in bacterial resistance. J Glob Antimicrob Resist. 2015;3(3):174-183. doi: 10.1016/j.jgar.2015.04.006. | No patient data from hospital information systems | CMA and DGA | November 2024 |
| 12 | Rajaonison A, Le Page S, Maurin T, Chaudet H, Raoult D, Baron SA, Rolain JM. Antilogic, a new supervised machine learning software for the automatic interpretation of antibiotic susceptibility testing in clinical microbiology: proof-of-concept on three frequently isolated bacterial species. Clin Microbiol Infect. 2022;28(9):1286.e1-1286.e8. doi: 10.1016/j.cmi.2022.03.035. | No patient data from hospital information systems | CMA and DGA | November 2024 |
| 13 | Chen Y, Chen X, Liang Z, Fan S, Gao X, Jia H, Li B, Shi L, Zhai A, Wu C. Epidemiology and prediction of multidrug-resistant bacteria based on hospital level. J Glob Antimicrob Resist. 2022;29:155-162. doi: 10.1016/j.jgar.2022.03.003. | No antimicrobial susceptibility tests | CMA and DGA | November 2024 |
| 14 | Feretzakis G, Loupelis E, Sakagianni A, Kalles D, Martsoukou M, Lada M, Skarmoutsou N, Christopoulos C, Valakis K, Velentza A, Petropoulou S, Michelidou S, Alexiou K. Using Machine Learning Techniques to Aid Empirical Antibiotic Therapy Decisions in the Intensive Care Unit of a General Hospital in Greece. Antibiotics (Basel). 2020;9(2):50. doi: 10.3390/antibiotics9020050. | No patient data from hospital information systems | CMA and DGA | November 2024 |
| 15 | Diéguez-Santana K, Casañola-Martin GM, Torres R, Rasulev B, Green JR, González-Díaz H. Machine Learning Study of Metabolic Networks vs ChEMBL Data of Antibacterial Compounds. Mol Pharm. 2022;19(7):2151-2163. doi: 10.1021/acs.molpharmaceut.2c00029. | No patient data from hospital information systems | CMA and DGA | November 2024 |
| 16 | Nocedo-Mena D, Cornelio C, Camacho-Corona MDR, Garza-González E, Waksman de Torres N, Arrasate S, Sotomayor N, Lete E, González-Díaz H. Modeling Antibacterial Activity with Machine Learning and Fusion of Chemical Structure Information with Microorganism Metabolic Networks. J Chem Inf Model. 2019;59(3):1109-1120. doi: 10.1021/acs.jcim.9b00034. | No patient data from hospital information systems | CMA and DGA | November 2024 |
