# Supplementary material for: Machine learning for predicting antimicrobial resistance in critical and high-priority pathogens: A systematic review considering antimicrobial susceptibility tests in real-world healthcare settings
Source: PLoS One. 2025 Feb 25;20(2):e0319460. doi: 10.1371/journal.pone.0319460 (PMC11856330; doi:10.1371/journal.pone.0319460)
Supplement: Attachment — Submitted filename: Responses.docx [file pone.0319460.s005.docx]

Dear Reviewers,

We are grateful for the constructive comments you provided, which helped us to improve the manuscript significantly.

Our responses to your comments are outlined below and highlighted in green in the new version.

***Reviewer #1:*** This is a well written report which provides a valuable contribution to avenues relevant to the exploration of AMR and AMS. Some sections require further clarification to provide context to the approach undertaken.

1. Line 56. Consider referencing the primary source rather than a secondary source.

**Response:** The primary resource was referenced following the recommendations.

2. Line 210. Aspects of data extraction is presented, however ‘personalised data extraction methods’ needs to be better explained.

**Response:** We thank the reviewer for this valuable comment. To address this, we have clarified the nature of the personalized data extraction methods used in our study. Specifically, we have detailed the development and use of customized and study-specific data extraction templates tailored to our research objectives. These templates facilitated the systematic collection of essential variables and publication-specific details, ensuring consistency and reliability across independent data collection efforts. The revised text can be found in Section 2.6 (Data Collection).

3. Line 228 to 231. A comprehensive narrative of the data analysis and reporting approach is needed.

**Response:** We appreciate the reviewer’s insightful suggestion. In response, we have expanded the description of our data analysis and reporting approach in Section 2.8 (Summary Measurements). Specifically, we have detailed the use of descriptive statistics for summarizing continuous outcomes and provided additional information on how we assessed heterogeneity to determine the feasibility of meta-analysis. Furthermore, we have clarified that in the absence of sufficient homogeneity, a qualitative synthesis was conducted to provide a narrative summary of the findings. This revision offers a more comprehensive explanation of our approach and addresses the reviewer's concern.

4. Line 244. Figure 1: A more detailed PRISMA flow chart is needed.

**Response:** We thank the reviewer for highlighting this point. In response, we have revised Figure 1 to include additional details for each stage of the study selection process. Considering that the study was submitted several months ago, we updated our search. We have detailed the records obtained in each database and provided clear descriptions of the reasons for exclusions during screening and full-text eligibility assessments, as well as the criteria used at each step. This enhanced figure offers a more transparent and detailed representation of the study selection methodology.

***Reviewer #2:***

1. This manuscript heavily discuses on AUROC. It would be helpful if the authors provided a figure that summarizes AUROC, and their conclusions based on the various articles they studied.

**Response:** A figure was provided following the recommendations.

Fig. 2. Heatmap of Area Under the Receiver Operating Characteristic Curve (AUROC) Values for Various Machine Learning Models Across Studies. This figure presents a heatmap summarizing the AUROC values reported in different studies for various machine learning models. Each cell represents the AUROC value reported in the corresponding study for the specific model, with darker colors indicating higher AUROC values (closer to 1.0), signifying better model performance. Blank cells indicate cases where no AUROC value was reported. Studies are arranged along the vertical axis, while machine learning models are organized along the horizontal axis. This heatmap provides a visual comparison of model performance across studies and highlights models that consistently achieve high AUROC scores.

2. Digital methods, such as machine learning, are undoubtedly valuable in various medical applications, as demonstrated in this study. However, these methods rely on the availability of computers and stable internet, which are often lacking in remote areas. In addition to outlining the benefits of this method, the authors should also address its limitations, particularly in areas with limited resources, such as developing countries. This is particularly important considering the author's statement that most deaths due to ARBs occur in developing countries (Line 60-61).

**Response:** The suggestion was recognized and the following text was added to the study limitations: Additionally, while digital methods such as machine learning have shown great promise in various medical applications, they rely heavily on access to computational resources and stable internet connectivity. This limitation is particularly significant in resource-limited settings, such as developing countries, where the burden of antimicrobial resistance and associated deaths is highest [3,4]. Future research should explore ways to adapt ML tools for offline use, develop lightweight algorithms that can operate on low-resource devices, or integrate these tools with existing healthcare systems in such regions.

3. Line 724: Is "metho" referring to a method?

**Response:** The typo was resolved.

***Reviewer #3:*** The manuscript is well-structured and tackles an important topic. However, it could be improved further by tackling the following:

1. Adding more actionable insights or suggestions for future research would further strengthen its contribution to the field.

**Response:** The suggestion was recognized and the following text was added to the study limitations: Future studies should aim to mitigate the impact of heterogeneity by employing strategies such as meta-regression or subgroup analyses to identify and address sources of variability. These methods, although not applied in the current review, could provide deeper insights into the influence of specific factors, such as study design, population characteristics, and ML algorithm choice, on outcomes.

Additionally, while digital methods such as machine learning have shown great promise in various medical applications, they rely heavily on access to computational resources and stable internet connectivity. This limitation is particularly significant in resource-limited settings, such as developing countries, where the burden of antimicrobial resistance and associated deaths is highest [3,4]. Future research should explore ways to adapt ML tools for offline use, develop lightweight algorithms that can operate on low-resource devices, or integrate these tools with existing healthcare systems in such regions.

Lastly, the study highlights the need for more actionable insights in future research. Specifically, efforts should focus on developing standardized reporting guidelines for ML studies to reduce variability, improving data quality and accessibility, and testing the scalability of ML models in real-world healthcare settings. Addressing these areas will enhance the practical applicability of ML in tackling AMR and other global health challenges.

2. You acknowledge the high heterogeneity across the included studies, which is appropriate. However, I recommend elaborating on how this heterogeneity might affect the generalizability of the results. Discussing possible strategies for reducing the impact of heterogeneity (e.g., meta-regression or subgroup analysis) would be useful, even if they weren’t applied in the current review.

**Response:** The suggestion was recognized and the following text was added to the study limitations:

The high heterogeneity observed in this study raises concerns about the generalizability of the results. Future studies should aim to mitigate the impact of heterogeneity by employing strategies such as meta-regression or subgroup analyses to identify and address sources of variability. These methods, although not applied in the current review, could provide deeper insights into the influence of specific factors, such as study design, population characteristics, and ML algorithm choice, on outcomes.

Additionally, while digital methods such as machine learning have shown great promise in various medical applications, they rely heavily on access to computational resources and stable internet connectivity. This limitation is particularly significant in resource-limited settings, such as developing countries, where the burden of antimicrobial resistance and associated deaths is highest [3,4]. Future research should explore ways to adapt ML tools for offline use, develop lightweight algorithms that can operate on low-resource devices, or integrate these tools with existing healthcare systems in such regions.

Lastly, the study highlights the need for more actionable insights in future research. Specifically, efforts should focus on developing standardized reporting guidelines for ML studies to reduce variability, improving data quality and accessibility, and testing the scalability of ML models in real-world healthcare settings. Addressing these areas will enhance the practical applicability of ML in tackling AMR and other global health challenges.

3. Consider grouping the results by key ML models or outcomes (e.g., by pathogens or prediction performance). This will help readers draw clearer connections between different studies.

**Response:** A figure was provided following the recommendations.

Fig. 2. Heatmap of Area Under the Receiver Operating Characteristic Curve (AUROC) Values for Various Machine Learning Models Across Studies. This figure presents a heatmap summarizing the AUROC values reported in different studies for various machine learning models. Each cell represents the AUROC value reported in the corresponding study for the specific model, with darker colors indicating higher AUROC values (closer to 1.0), signifying better model performance. Blank cells indicate cases where no AUROC value was reported. Studies are arranged along the vertical axis, while machine learning models are organized along the horizontal axis. This heatmap provides a visual comparison of model performance across studies and highlights models that consistently achieve high AUROC scores.
