# Supplementary material for: Machine learning for predicting antimicrobial resistance in critical and high-priority pathogens: A systematic review considering antimicrobial susceptibility tests in real-world healthcare settings
Source: PLoS One. 2025 Feb 25;20(2):e0319460. doi: 10.1371/journal.pone.0319460 (PMC11856330; doi:10.1371/journal.pone.0319460)
Supplement: Attachment — Submitted filename: Responses Reviewer 3.docx [file pone.0319460.s006.docx]

Dear Reviewer,

Our responses to your comments are outlined below and highlighted in green in the new version.

Reviewer #3:

1. The introduction presents interesting statistics on the estimated number of infections and deaths, amongst others. It would be helpful if the authors stated clearly in each instance of the statistics whether it is global or related to developing countries.

**Response:** We have carefully reviewed the introduction and incorporated the suggested clarifications. In the revised version of the manuscript, we have explicitly stated that the statistics cited are based on global data. For example:

The projected number of deaths from bacterial infections by 2050 is described as occurring on a global scale.

The data from 2019 on deaths linked to antibiotic-resistant bacteria (ARBs) is specified as worldwide.

The statistics on methicillin-resistant *S. aureus* (MRSA) and other multidrug-resistant pathogens are clarified as global or occurring across the globe.

2. Authors should reference and discuss previous systematic literature reviews in the domain and make a case for the relevance of the current review. Below are some examples of related reviews in the domain:

a) Lv, G., & Wang, Y. (2024). Machine learning-based antibiotic resistance prediction models: An updated systematic review and meta-analysis. Technology and Health Care, (Preprint), 1-18.

b) Sakagianni, A., Koufopoulou, C., Feretzakis, G., Kalles, D., Verykios, V. S., & Myrianthefs, P. (2023). Using machine learning to predict antimicrobial resistance―a literature review. Antibiotics, 12(3), 452.

c) E. Elyan, A. Hussain, A. Sheikh, A. A. Elmanama, P. Vuttipittayamongkol and K. Hijazi, "Antimicrobial Resistance and Machine Learning: Challenges and Opportunities," in IEEE Access, vol. 10, pp. 31561-31577, 2022, doi: 10.1109/ACCESS.2022.3160213.

**Response:** Thank you for your thoughtful suggestion to include additional systematic literature reviews in our manuscript to further support the relevance of our current review. We appreciate the references provided and acknowledge their contributions to the field. However, we would like to respectfully clarify that our systematic review already incorporates and discusses several relevant and high-impact systematic reviews and meta-analyses addressing antimicrobial resistance (AMR) and machine learning-based prediction models. These include:

- O’Neill J (2016). Tackling Drug-Resistant Infections Globally.

- Antimicrobial Resistance Collaborators. Global burden of bacterial antimicrobial resistance in 2019: a systematic analysis. Lancet. 2022;399(10325):629-655. doi: 10.1016/S0140-6736(21)02724-0

- Tang et al. (2022) – A systematic review and meta-analysis on machine learning in predicting antimicrobial resistance [39].

- Pormohammad et al. (2019) – A systematic review and meta-analysis on antibiotic resistance in *E. coli* strains isolated from multiple sources [6].

- Christodoulou et al. (2019) – A systematic review evaluating machine learning versus logistic regression for clinical prediction models [43].

- Beunza et al. (2019) – A comparison of machine learning algorithms for predicting clinical events, [44].

- Sufriyana et al. (2020) – A systematic review and meta-analysis comparing multivariable logistic regression with machine learning algorithms [45].

- Delpino et al. (2022) – A systematic review on machine learning applications for predicting chronic diseases [52].

Additionally, we have included foundational reviews such as O’Neill (2016) [2] and the Antimicrobial Resistance Collaborators (2022) [3], which provide a broader context of AMR's global impact and underscore the urgency of predictive approaches.

Given the comprehensive nature of these cited works, we believe our manuscript already provides a well-rounded background without overwhelming the reader with excessive references.

3. The author identifies data imbalance as one of the issues with existing data in the domain. Authors can, therefore, consider referencing the following article:

Brown, S. A., Weyori, B. A., Adekoya, A. F., Kudjo, P. K., & Mensah, S. (2022). Predicting Blocking Bugs with Machine Learning Techniques: A Systematic Review. International Journal of Advanced Computer Science and Applications, 13(6).

**Response:** We appreciate your insightful suggestion to include the recommended reference by Brown et al. (2022) to strengthen our discussion on data imbalance challenges in machine learning applications. In response, we have incorporated the reference into the discussion section of our revised manuscript. Specifically, we highlight how the study by Brown et al. underscores the impact of imbalanced datasets on evaluation metrics and emphasizes the need for more robust validation approaches. This addition provides a broader perspective on addressing data imbalance issues across domains involving machine learning models.

4. Authors should suggest standardizing ML study reporting in AMR research, given the gaps identified in this work.

**Response:** We have revised the discussion section to explicitly emphasize the importance of developing and adopting standardized reporting guidelines for ML studies. This addition highlights the necessity of reducing variability, ensuring methodological transparency, and facilitating reproducibility in future AMR research.


5. Authors should proofread their work thoroughly to correct grammatical errors.

**Response:** A thorough review of the manuscript was conducted, and grammatical errors were corrected.
