# Supplementary material for: Machine learning for predicting antimicrobial resistance in critical and high-priority pathogens: A systematic review considering antimicrobial susceptibility tests in real-world healthcare settings
Source: PLoS One. 2025 Feb 25;20(2):e0319460. doi: 10.1371/journal.pone.0319460 (PMC11856330; doi:10.1371/journal.pone.0319460)
Supplement: Attachment — Submitted filename: PONE D 24 19315 Reviewer comments.docx [file pone.0319460.s003.docx]

Line 56

Consider referencing the primary source rather than a secondary source.

Line 210

Aspects of data extraction is presented, however ‘personalised data extraction methods’ needs to be better explained.

Line 228 to 231

A comprehensive narrative of the data analysis and reporting approach is needed.

Line 244

Figure 1: A more detailed PRISMA flow chart is needed.
